# Pituitary Phenotypes of Mice Lacking the Notch Signalling Ligand Delta-Like 1 Homologue

**DOI:** 10.1111/jne.12010

**Published:** 2013-03-21

**Authors:** L Y M Cheung, K Rizzoti, R Lovell-Badge, P R Tissier

**Affiliations:** * Divisions of Molecular Neuroendocrinology and Developmental Genetics and Stem Cell Biology, MRC National Institute for Medical ResearchLondon, UK; † Neural Development Unit, Institute of Child Health, University College LondonLondon, UK

**Keywords:** growth hormone, pituitary, Dlk1, Pref1

## Abstract

The Notch signalling pathway ligand delta-like 1 homologue (Dlk1, also named Pref1) is expressed throughout the developing pituitary and becomes restricted to mostly growth hormone (GH) cells within the adult gland. We have investigated the role of Dlk1 in pituitary development and function from late embryogenesis to adulthood using a mouse model completely lacking the expression of Dlk1. We confirm that Dlk1-null mice are shorter and weigh less than wild-type littermates from late gestation, at parturition and in adulthood. A loss of Dlk1 leads to significant reduction in GH content throughout life, whereas other pituitary hormones are reduced to varying degrees depending on sex and age. Both the size of the pituitary and the proportion of hormone-producing cell populations are unchanged, suggesting that there is a reduction in hormone content per cell. *In vivo* challenge of mutant and wild-type littermates with growth hormone-releasing hormone and growth hormone-releasing hexapeptide shows that reduced GH secretion is unlikely to account for the reduced growth of Dlk1 knockout animals. These data suggest that loss of Dlk1 gives rise to minor pituitary defects manifesting as an age- and sex-dependent reduction in pituitary hormone contents. However, Dlk1 expression in other tissue is most likely responsible for the weight and length differences observed in mutant animals.

The anterior pituitary gland has a central role in physiology, regulating various processes such as growth, lactation and pregnancy, reproduction, metabolism, and response to stress. The five different hormone-producing cell types within the gland are specified during embryogenesis by a cascade of transcription factors and signalling pathways 1, although the number of cells of each population increases after birth 2 and can vary throughout life in response to physiological demand 3. These changes in cell number and function are under the control of hypothalamic and peripheral signals 3,4, although paracrine and autocrine factors are also considered to be involved 5. Paracrine factors are likely to regulate differentiation of the recently described multipotent progenitor, or stem cells, of the pituitary 6, especially for cell types such as somatotrophs (producing growth hormone; GH), where the receptor for the primary proliferative signal is restricted to the differentiated cell type 7.

The Notch pathway is a conserved signal transduction mechanism first identified in *Drosophila* 8 that is activated by the binding of a transmembrane ligand to the transmembrane Notch receptor on a neighbouring cell. This interaction results in cleavage of the receptor to form the Notch intracellular domain, which translocates to the nucleus, binds RBPjκ and initiates transcription of target genes such as Hes1 and Hey1 9. Various components of the Notch pathway are expressed during pituitary development, including the Notch2 and Notch3 receptors, the ligand Jagged1, and the downstream effector Hes1 10,11. Overexpression of Hes1 in differentiating pituitary cells *in vivo* has been shown to inhibit gonadotroph and thyrotroph differentiation in mice 12. Conversely, Hes1-deficient mice display increased cell cycle exit and increased expression of cyclin-dependent kinase inhibitors such as p27 in the pituitary 13, whereas Hes1 and Prop1 double-mutants show premature differentiation of corticotrophs 14. Persistent expression of the receptor Notch2 during embryogenesis causes a reduction in the number of thyrotrophs and delays gonadotroph differentiation, although the gonadotroph population is rescued as the mice develop to maturity 15. Conditional deletion of the Notch effector RBPjκ in the developing mouse embryo leads to premature differentiation of corticotrophs and, conversely, overexpression of the active Notch receptor inhibits terminal differentiation 11. Taken together, this evidence points towards Notch signalling as a regulator of differentiation timing within pituitary hormone cell types.

The nonclassical ligand delta-like 1 homologue (Dlk1), a paternally-imprinted gene on mouse chromosome 12 16, is expressed throughout the developing Rathke's pouch from embryonic day (E) 10.5 17 and in the adult anterior pituitary, as well as in bone, β-cells in pancreatic islets, placenta and adrenal glands 17–21. The protein is expressed in the majority of GH cells in the pituitary gland, and a low proportion of all other hormone cell types 22,23, as well as the Sox2-expressing putative stem/progenitor cells, which can form pituispheres in culture 24,25. *In vitro* studies have previously shown that, in somatolactotroph GH3 cells overexpressing Dlk1, GH expression and secretion are down-regulated 26. Expression of Dlk1 is also increased in human hormone-secreting pituitary tumours 27, whereas silencing of the Dlk1/MEG3 imprinted locus is detected in nonfunctioning pituitary adenomas 28,29. This pattern of expression suggests a role for Dlk1 in normal pituitary development and function.

One of the observed phenotypes of mice lacking Dlk1, generated by deletion of exons 2 and 3, is growth retardation 30, which was later confirmed in a similar but independently-generated Dlk1-null mutant deleting the promoter and exons 1–3 31. A recent study using a mouse model with altered expression of several imprinted genes, including overexpression of Dlk1, reported a reduced weight of transgenics at weaning associated with a failure to thrive 32. Therefore, either increased or decreased expression of the Dlk1 gene may have an effect on the growth of the mice. A recent study using the Dlk1-null mutant generated by Raghunandan *et al*. 31 found fewer GH-, prolactin (PRL)- and follicle-stimulating hormone (FSH)-immunoreactive cells in the adult pituitary, and an increased serum leptin concentration 23. Interestingly, the organisation of somatotrophs, an important factor in robust GH secretion 33, was found to be altered, suggesting that the growth phenotype in null animals may be caused in part by an altered GH axis. In the present study, we have expanded on these previous studies by assessing the effect of loss of Dlk1 on growth and pituitary hormones, in particular investigating whether the growth retardation could be accounted for by a deficient secretion of GH.

## Materials and methods

### Genetically-modified mice strains

We have characterised pituitary phenotypes in previously-generated Dlk1-null mutant mice 31 on a C57BL/6J background. Genomic DNA from ear biopsies was used for polymerase chain reaction genotyping using previously described primers. Because Dlk1 is a paternally-imprinted gene 16,17,34, we compared wild-type with *Dlk1*^*+/−Pat*^ mice, with the null allele paternally inherited (referred to as Dlk1-null mice), except when comparing heterozygotes with homozygous null mice. Mutants show no noticeable impairment in fertility and litter size.

### *In vivo* experiments

Mice were given access to water and chow *ad lib*., and experiments were performed in accordance with Institutional and Home Office legislation and guidelines. Weights were recorded weekly between age-matched littermates after weaning at 3 weeks of age. Body lengths were measured after mice were sacrificed. Pituitary response to acute challenge by GH secretagogues was performed as described previously 35.

### Radioimmunoassays

Total pituitary hormone contents were assayed using a previously described method 36 using mouse-specific reagents kindly provided by A. L. Parlow [National Hormone and Pituitary Program (NHPP), Torrance, CA, USA].

### Cell dispersion

Pituitary glands were dispersed as previously described 37 and all cells plated onto 13-mm diameter coverslips coated with polylysine (Sigma, St Louis, MO, USA). Cell counts of dispersed cells were performed manually after immunofluorescence imaging.

### Immunofluorescence and microscopy

Pituitaries were perfusion-fixed with 4% w/v paraformaldehyde in phosphate-buffered saline (PBS), and cryosectioned at 12 μm. Sections or dispersed cells were blocked with blocking solution (10% w/v donkey serum in PBS/0.1% Triton X-100; PBST), and then used for immunohistochemistry with overnight incubation at 4 °C using primary antibodies in 10% blocking solution at the dilutions: monkey anti-rat GH (NHPP) at 1 : 5000; rabbit anti-mouse prolactin (a gift from Professor F. Talamantes, University of Santa Cruz, CA, USA) at 1 : 10 000; rabbit anti-mouse luteinising hormone (LH) (NHPP) at 1 : 1000; rabbit anti-adrenocorticotrophic hormone (ACTH) (NHPP) at 1 : 500; guinea pig anti-thyroid-stimulating hormone (TSH) (NHPP) at 1 : 50; rabbit anti-mouse Dlk1 (Santa Cruz Biotechnology, Santa Cruz, CA, USA) at 1 : 100; goat anti-Sox2 (Immune Systems Limited, Paignton, UK) at 1 : 500; rat anti-platelet endothelial cell adhesion molecule-1 (PECAM-1) at 1 : 50 (BD Pharmingen, San Diego, CA, USA). After washing in PBST, sections or coverslips were incubated at room temperature for 1 h with the appropriate anti-rabbit secondary antibody conjugated to Alexa-488, or -555, and anti-human Alexa-594, in blocking solution with 1 μm 4′,6-diamidino-2-phenylindole (DAPI). After washing in PBST, sections or coverslips were mounted with Aqua-Poly/Mount (Polysciences, Inc., Warrington, PA, USA). The fluorescent signal was visualised using fluorescent and confocal microscopes (TCS SP5 and SPE; Leica Microsystems, Wetzlar, Germany) using ×63 or ×100 oil immersion objectives.

### Total pituitary protein measurement

Total protein from pituitary samples was measured using the Micro BCA™ Protein Assay Reagent Kit (Thermo Scientific Pierce, Rockford, IL, USA) in accordance with the manufacturer's instructions.

### Statistical analysis

Quantitative data are shown as the mean ± SEM. Differences between groups were analysed by an unpaired Student's t-test. Analyses of growth curves was analysed using a linear mixed-effects model of weight versus age using genotype as fixed factors and subjects (mice) as random factors, with anova to test the overall effect of genotype on growth followed by Tukey's post-hoc tests.

## Results

### Loss of Dlk1 expression leads to growth retardation

**Fig. 1:**
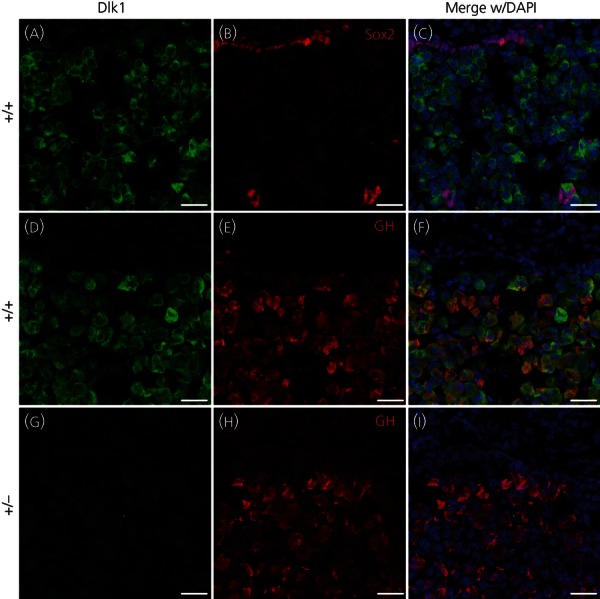
Immunohistochemistry for delta-like 1 homologue (Dlk1) and growth hormone (GH) in the pituitaries of Dlk1-null mice and wild-type littermates. Sections from pituitaries of male wild-type (a–f) and Dlk1-null mutant (g–i) mice stained for Dlk1 (green), Sox2 or GH (red) and 4′,6-diamidino-2-phenylindole (DAPI) (blue). The posterior lobe is orientated at the top of the image, and the anterior at the bottom. Dlk1 is mostly excluded from Sox2-positive cells (a–c), although it is expressed in the majority of somatotrophs in the anterior pituitary (d–f), as previously shown. Expression of Dlk1 is completely lacking in Dlk1-null mutants in the pituitary gland (g–i). Scale bar = 20 μm.

**Fig. 2:**
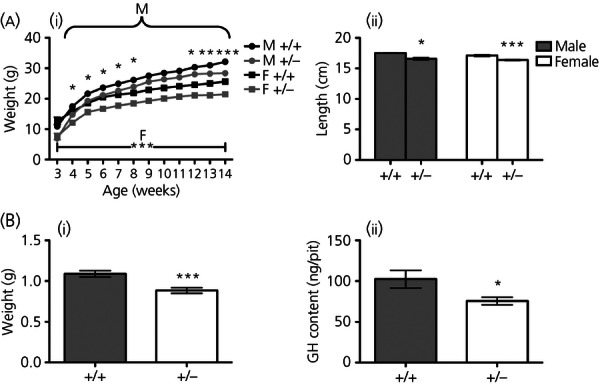
Growth differences between delta-like 1 homologue (Dlk1)-null mutant and wild-type littermate mice and analyses of Dlk1-null mutant in late embryonic development. (a, i) Weights of male and female wild-type and Dlk1-null mutant mice from weaning at 3 weeks to 14 weeks of age. Males have a statistically significantly reduced weight at the majority of timepoints, and females have a lower weight from weaning to 14 weeks of age, although their rate of growth appears normal. (a, ii) Nose-to-tail measurements of the body length of wild-type and Dlk1-null mutant mice at 14 weeks of age. (b, i) Weights of E18.5 mutant embryos and their wild-type counterparts at E18.5. (b, ii) Pituitaries of Dlk1-null mutant mice and wild-type littermates were assayed for growth hormone (GH) content. (a) Data shown are the mean ± SEM of 7–10 mice. (b) Data shown are the mean ± SEM of 12–13 mice. Statistical analysis by mixed-effects model of weight versus age using genotype as fixed factors and subjects (mice) as random factors, with anova to test the overall effect of genotype on growth followed by Tukey's post-hoc tests (a) and unpaired t-test (b). *P < 0.05; **P < 0.01; ***P < 0.001 Dlk1-null versus wild-type littermates.

Immunohistochemistry for Dlk1 confirmed expression in normal pituitary, a loss of expression in Dlk1 null animals, as well as expression in somatotrophs in wild-type animals, with some nonsomatotroph cells also expressing the protein, although the majority of Sox2-positive cells do not express Dlk1 (Fig. 1). As reported previously 30, a loss of Dlk1 led to a visible reduction in body size. Weight measurements beginning at 3 weeks of age demonstrated that males have a significantly reduced weight at the majority of timepoints, and females had a lower weight from weaning to 14 weeks of age (Fig. 2a, i). However, there is no difference in the weight gained between wild-types and mutants between 4 and 14 weeks of age (weight gained between 4 and 14 weeks of age: males +/+14.73 ± 0.67 g versus +/−13.64 ± 0.75 g; females +/+9.91 ± 0.58 g versus +/−9.34 ± 0.71 g; n = 7–10; wild-type versus heterozygote not significant), showing that the rate of growth of the mutants post-weaning is normal. The nose-tail length of Dlk1-null mice at 14 weeks of age was reduced compared to wild-type littermates in both male and female mice (Fig. 2a, ii), consistent with them having a reduction in body size from birth without subsequent catch-up growth. Embryos at E18.5 were lighter than wild-type littermates (Fig. 2b, i) and also had a reduced pituitary GH content (Fig. 2b, ii).

Weight monitoring, length measurements and hormone content measurements from paternally-inherited Dlk1 heterozygotes and homozygous null mice were also recorded to confirm whether reactivation of the maternal wild-type allele occurred as mice grew past adolescence. Heterozygotes show the same weights and growth rate as homozygotes from 3–6 weeks of age (weight at 6 weeks: males +/−20.52 ± 0.21 g versus −/−20.44 g ± 0.56 g; females +/−16.70 ± 0.31 g versus −/−17.22 ± 0.38 g; n = 3–6; heterozygote versus homozygote not significant), and no differences in body lengths at 6 weeks of age (nose-to-tail lengths: males +/−15.46 ± 0.11 cm versus −/−15.40 ± 0.15 cm; females +/−14.68 ± 0.13 cm versus −/−14.84 ± 0.11 cm; n = 5–6; heterozygote versus homozygote not significant).

### Dlk1-null mutants have age- and sex-specific reductions in all pituitary hormones

Pituitaries from E18.5 embryos and 2-, 6- and 14-week-old mice were dissected for total pituitary hormone content measurements by radioimmunoassay. A loss of Dlk1 led to a 30% reduction in pituitary GH content in E18.5 embryos, which remained reduced in both male and female mutants (Fig. 2b, ii), from juveniles (2 weeks) to fully-adult mice (14 weeks) (Table 1).

**Table 1:** Post-Natal Pituitary Hormone Content of Delta-Like 1 Homologue (Dlk1)-Null Mutant and Wild-Type Littermates Over Time

Hormone	P14 +/+	P14 +/−	P42 +/+	P42 +/−	P100 +/+	P100 +/−
Male						
GH	4.03 μg ± 0.45	2.81 μg ± 0.28(*)	63.32 μg ± 3.51	47.21 μg ± 3.32(**)	77.58 μg ± 4.84	52.63 ug ± 2.80(***)
PRL	8.99 ng ± 1.26	6.99 ng ± 1.42 (NS)	0.33 μg ± 0.10	0.24 μg ± 0.035 (NS)	1.67 μg ± 0.31	1.41 μg ± 0.11 (NS)
TSH	0.37 μg ± 0.027	0.28 μg ± 0.0091 (**)	0.44 μg ± 0.014	0.34 μg ± 0.030 (**)	0.56 μg ± 0.036	0.39 μg ± 0.019(**)
ACTH	7.67 ng ± 0.75	5.77 ng ± 0.71 (NS)	69.44 ng ± 4.45	54.63 ng ± 2.80 (*)	145 ng ± 13.70	115 ng ± 13.36 (NS)
LH	45.71 ng ± 5.15	21.0 ng ± 2.95(**)	2.28 μg ± 0.17	1.49 μg ± 0.19	1.25 μg ± 0.075	0.81 μg ± 0.048(***)
FSH	7.76 ng ± 0.75	5.77 ng ± 0.71 (NS)	287 ng ± 23.35	172 ng ± 17.10(**)	536 ng ± 35.91	352 ng ± 25.58(**)
Females						
GH	6.658 μg ± 1.00	2.575 μg ± 0.28(**)	35.23 μg ± 3.98	20.21 μg ± 2.22 (*)	80.76 μg ± 4.43	55.11 μg ± 5.83(**)
PRL	27.17 ng ± 6.10	11.04 ng ± 1.35(*)	1.69 ng ± 0.20	0.88 μg ± 0.27(*)	4.61 ng ± 0.64	6.18 ng ± 0.80(NS)
TSH	0.37 μg ± 0.031	0.30 μg ± 0.027 (NS)	0.37 μg ± 0.025	0.24 μg ± 0.025 (**)	0.53 μg ± 0.013	0.39 μg ± 0.019 (***)
ACTH	7.36 ng ± 1.17	4.18 ng ± 0.81(*)	58.94 ng ± 2.85	49.88 ng ± 3.20(NS)	93.98 ng ± 3.17	113.4 ng ± 9.75 (NS)
LH	107.6 ng ± 24.3	28.1 ng ± 2.78 (**)	0.31 μg ± 0.049	0.25 μg ± 0.081 (NS)	0.41 μg ± 0.36	0.30 μg ± 0.036 (*)
FSH	7.36 ng ± 1.17	4.18 ng ± 0.81 (**)	20.83 ng ± 1.82	12.89 ng ± 2.92(*)	33.11 ng ± 2.58	23.56 ng ± 1.30(**)

Pituitaries from 2-, 6- and 14-week old Dlk-1 null mutant and wild-type were assayed for GH, prolactin (PRL), thyroid-stimulating hormone (TSH), adrenocorticotrophic hormone (ACTH), luteinising hormone (LH) and follicle-stimulating hormone (FSH). All hormones show a mild reduction in content at some time during the mice's life, although not all remain deficient. Data are shown as the mean ± SEM of seven to ten mice. *P < 0.05; **P < 0.01; ***P < 0.001 Dlk1-null versus wild-type littermates. NS, not significant.

**Fig. 3:**
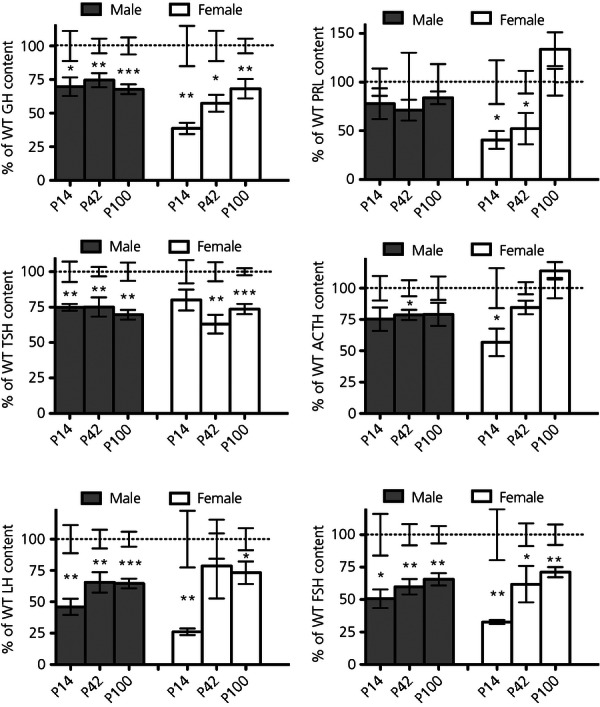
Post-natal progression of pituitary hormone defects in delta-like 1 homologue (Dlk1)-null mutants expressed as percentage of wild-type (WT) littermates. The data in Table 1 expressed as a percentage of the corresponding wild-type content at 2, 6 and 14 weeks of age. Data are shown as the mean ± SEM of seven to ten mice. *P < 0.05; **P < 0.01; ***P < 0.001 Dlk1-null versus wild-type littermates. ACTH, adrenocorticotrophic hormone; FSH, follicle-stimulating hormone; GH, growth hormone; LH, lutesinsing hormone; PRL, prolactin; TSH, thyroid-stimulating hormone.

Other pituitary hormones are also reduced to varying extents in the mutants in an age- and sex-dependent manner. Mutant hormone contents expressed as a percentage of the wild-type content show the progression of hormone reduction phenotypes over time (Fig. 3). GH and FSH contents are reduced in both sexes at all three ages; PRL content is reduced in 2- and 6-week-old females; TSH content is significantly reduced at all time-points, except at 2 weeks of age in females; ACTH content is reduced at 6 weeks of age in males and at 2 weeks of age in females; and LH contents are reduced at all time-points except 6 weeks of age in females. PRL and ACTH contents in Dlk1-null mutant females return to normal by 14 weeks of age. There was no difference in the pituitary hormone contents of homozygous null animals compared to paternally-inherited Dlk1 heterozygote littermates at 6 weeks of age (total GH content per pituitary: males +/−67.08 ± 8.18 μg versus −/−68.10 ± 7.87 μg; females +/−34.79 ± 3.75 μg versus −/−37.90 ± 3.40 μg; n = 5–6; heterozygote versus homozygote not significant).

To address the possibility that the Dlk1-null mutants have reduced pituitary hormone content simply as a result of a smaller pituitary gland, we measured total pituitary protein content in wild-type and Dlk1-null male and female mice at 6 weeks of age. There was no significant reduction in the total protein content of the Dlk1-null mice compared to wild-type littermates (males +/+0.90 ± 0.053 μg versus +/−0.84 ± 0.033 μg; females +/+0.81 ± 0.041 μg versus +/−0.73 ± 0.037 μg, n = 4–9, wild-type versus heterozygote not significant).

### Proportions of cell populations unchanged in Dlk1-null female mutants

**Fig. 4:**
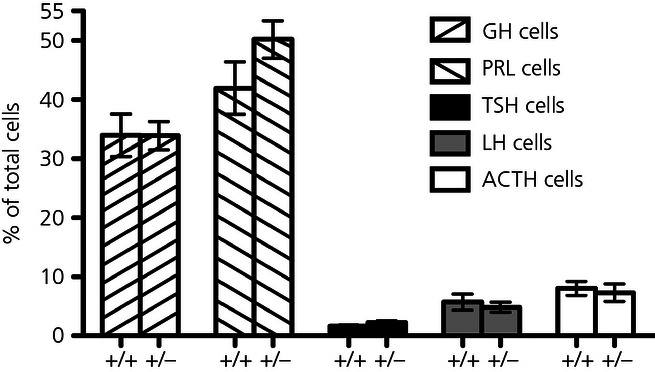
Proportion of hormone cell populations in female wild-type and delta-like 1 homologue (Dlk1)-null mutant mice. Pituitaries from 6-week-old female Dlk1-null and wild-type mice were dispersed and the number of cells positive for each pituitary hormone quantified after immunofluorescent staining. Data are shown as the mean ± SEM of three to six mice. ACTH, adrenocorticotrophic hormone; FSH, follicle-stimulating hormone; LH, lutesinsing hormone; PRL, prolactin; TSH, thyroid-stimulating hormone.

A reduction in pituitary hormone content could result from a reduced number of hormone-producing cells. To determine whether this was the case after loss of Dlk1, pituitaries from 6-week-old female wild-type and null mutants were dispersed and plated for immunohistochemistry for individual hormones, allowing for imaging and manual quantification of hormone populations. No significant differences that could account for the reduction in hormone content was observed in any of the hormone-producing cell populations in the mutant mice (Fig. 4). There was no evidence for adenoma formation in pituitaries, either at this age or in the pituitaries of 14-week-old animals.

### Minor defect in sustained GH secretion in Dlk1-null male mutants

**Fig. 5:**
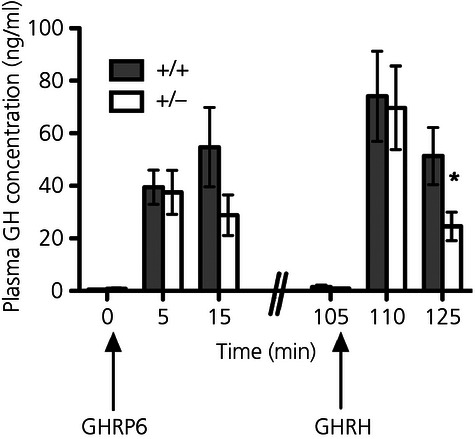
Comparison of gowth hormone (GH) release in response to challenge in delta-like 1 homologue (Dlk1)-null and wild-type mice. Jugular catheters were inserted into anaesthetised male wild-type and Dlk1-null mutant mice, and blood samples taken before and 5 and 15 min after i.v. injection of 500 ng of growth hormone-releasing hexapeptide (GHRP-6). Ninety minutes later, blood samples were taken before and 5 and 15 min after i.v. injection of 100 ng growth hormone-releasing hormone (GHRH). Plasma was isolated and assayed for circulating GH by radioimmunoassay. Data are shown as the mean ± SEM of six to eight mice. *P < 0.05 Dlk1-null versus wild-type littermates.

Six-week-old male wild-type and Dlk1-null mutants were assessed for their ability to secrete GH in response to the GH secretagogues growth hormone-releasing hormone (GHRH) and growth hormone-releasing hexapeptide (GHRP-6) (Fig. 5). Basal plasma GH concentrations before the administration of secretagogues were low in both wild-type and mutant animals, as expected. Both GHRP-6 and GHRH caused GH secretion into the circulation in both wild-type and mutant mice. The GH response in mutants is normal at all time-points except one, with the only significant reduction observed being at 15 min after administration of the second secretagogue (GHRH).

### Dlk1 in the placental labyrinth and its role in intrauterine growth restriction (IUGR)

**Fig. 6:**
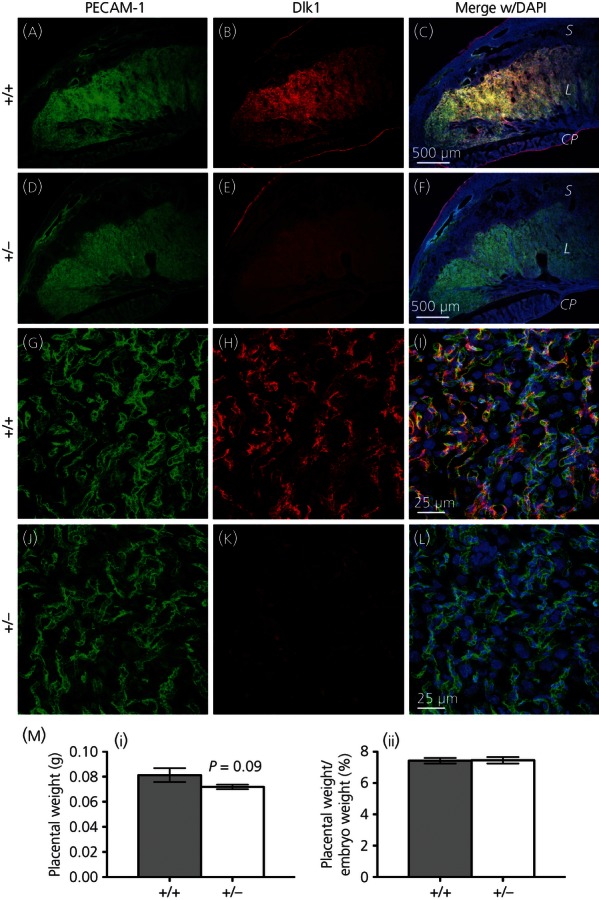
Embryonic development of the placenta in the delta-like 1 homologue (Dlk1)-null mutant. Sections from placentas of wild-type and Dlk1-null mutant E18.5 embryos stained for platelet endothelial cell adhesion molecule-1 (PECAM-1) (green), Dlk1 (red) and 4′,6-diamidino-2-phenylindole (DAPI) (blue). Expression of Dlk1 in the placenta is predominantly in the labyrinth, where PECAM-1 is expressed in a large number of foetal blood vessels (a–c); although Dlk1 expression is lacking in the mutant embryo, the foetal labyrinth appears to have formed normally (d–f). *S*, spongiotrophoblast*; L*, labyrinth*; CP*, chorionic plate. Low magnification scale bar = 500 μm. Dlk1 colocalises with many of the PECAM-1-expressing endothelial cells in the wild-type embryo (g–i). The expression of PECAM-1 is not affected by the loss of Dlk1 in the mutant embryo (j–l). High magnification scale bar = 25 μm. (m, i) Placental weights of Dlk1-null mutants are slightly, but not significantly (Dlk1-null versus wild-type littermates), reduced at E18.5. (m, ii) Placental weights expressed as a percentage of the embryo weight shows no difference between the wild-type and Dlk1-null embryos, showing that placental weights are reduced to the same extent as the embryo weights. Data are shown as the mean ± SEM of three to five embryos.

Because Dlk1 mutant neonates have a reduced weight at birth and Dlk1 has previously been shown to be expressed in the embryonic vessels of the placenta, we examined the placentas of Dlk1 mutant mice for any obvious aetiologies for intrauterine growth restriction. Immunohistochemistry of placentas of wild-type mice at E18.5 confirmed the expression of Dlk1 in foetal endothelia, shown by its colocalisation with platelet endothelial cell adhesion molecule-1 (PECAM-1) (Fig. 6a–c). Dlk1 is not expressed in the placental labyrinth of Dlk1 mutant embryos, as expected, although there is no obvious alteration in PECAM immunostaining, indicating normal foetal vessel formation (Fig. 6d–f). Higher magnification of the labyrinth of mutant animals shows there is no overt defect in the branching of the trophoblast layer and the formation of the foetal endothelial cells (Fig. 6g–l). Placental weights were also measured at E18.5 to study placental insufficiency as a cause of IUGR. Although it appears that placentas of Dlk-1 null animals may have a reduced weight compared to wild-type littermates (P = 0.09) (Fig. 6m, i), expressing this as a percentage of the embryo weight shows a proportional placental weight reduction (Fig. 6m, ii).

## Discussion

The present study investigated physiological and pituitary phenotypes in a previously-generated Dlk1-null mutant mouse strain 31. Although initially generated for a study on B lymphocytes, Raghunandan *et al*. (31) also noted the growth phenotype of the null mutants. A recent study using this mouse model investigated the cellular and subcellular localisation of Dlk1 within hormone-producing cells, and the effect of its loss within those cells 23, finding an effect on the somatotroph, lactotroph and gonadotroph axes, and elevated circulating leptin concentrations. In the present study, we have shown that a loss of Dlk1 leads to effects on all pituitary hormones, although the reduction in GH secretion is unlikely to cause the size reduction associated with a loss of Dlk1.

We have confirmed the paternal expression of Dlk1 in the pituitary because the inheritance of a normal maternal allele and a null paternal allele led to a loss of pituitary expression, with the antigen of the antibody in an unaltered part of the Dlk1 protein. We also show that the majority of Sox2-positive cells do not express Dlk1, although they have been reported to be co-expressed in the same cell population 24. It has previously been reported that certain genetic and epigenetic factors may cause reactivation of the silent maternal allele of Dlk1 in the brain 38 and other imprinted genes have been shown to undergo post-natal loss of imprinting 39; however, at 6 weeks of age, we could not detect any reactivation in the pituitary. To confirm that reactivation in other tissues or at other ages was not affecting the phenotypes analysed, we also compared homozygous Dlk1-null animals with heterozygous animals with a paternally inherited null allele. There was no difference in weight, growth rate, lengths or any hormone contents between the heterozygous and the homozygous null mice at 6 weeks of age, allowing us to use heterozygotes with a paternally-inherited null allele as functionally null mutants in all our subsequent studies.

It has been previously reported that the Dlk1 null-mutant mice generated by Moon *et al*. (30) are visibly smaller compared to age-matched littermates and weighed less from 20 to 120 days of age. Monitoring of the weight of the Dlk1-null mutants used [from Raghunandan *et al*. 31] from weaning at 3 weeks of age until 14 weeks of age shows a reduced weight throughout life but a normal rate of growth. The mutant mice continue to have reduced weight compared to wild-type littermates as they become older. This differs to the growth phenotype described for the Dlk1-null mutant strain generated by Moon *et al*. (30) where mutants eventually return to weights similar to those of wild-type animals. However, the Dlk1-null strain generated by Moon *et al*. (30) deletes a larger portion of the Dlk1 gene and the background strain was BALB/cJ 27, which may lead to subtle differences in phenotype. Background effects on Dlk1-null phenotypes were previously identified by Moon *et al*. 30 with an eyelid defect on a BALB/cJ background that is absent on a C57BL/6 background. This may also be the cause of phenotypic differences between the present study and those observed by both Raghunandan *et al*. 31 and Puertas-Avendano *et al*. 23, who used the Dlk1-null strain on a 129/SvJ background.

The length of Dlk1-null mutants was reduced compared to wild-type littermates, similar to those found in a mouse strain with a low GH content as a result of targeted ablation of GH cells 40. GH deficiency would be consistent with the post-weaning weight and length reductions of Dlk1-null mutants but does not account for the reduced size of E18.5 embryos because other mice with more severe GH deficiency do not show any size difference at this age 41. However, we are unable to exclude a mechanism whereby a combination of a loss of Dlk1 with GH deficiency could cause growth retardation in the embryo. It is also unlikely that any effects on body size at older ages are simply a result of reduced pituitary GH content because other models with a similar reduction have normal body length and weight from weaning to 14 weeks. Because total pituitary GH content is far in excess of the amount secreted per pulse, a mild reduction in total GH protein may be insufficient to cause a growth defect.

The reduction in weight of Dlk1-null embryos at E18.5 compared to their wild-type littermates suggests a role for Dlk1 in intrauterine growth retardation (IUGR). Dlk1 is expressed in the endothelial cells of the placental labyrinth 21, the interface between the maternal and foetal circulations that allows nutrient exchange with the foetal vasculature 42, prompting us to determine whether the altered function of these endothelial cells in Dlk1-null animals may be resonsible for the IUGR. In the Dlk1 mutant embryos at E18.5, the expression of Dlk1 found in the placenta of wild-type embryos is lost but endothelial cells are able to form normally and there is no observed defect in vascular expression of PECAM-1. This suggests that Dlk1 is not required for branching morphogenesis of the trophoblast layer that forms the placental labyrinth. The ratio of the placental weight to the embryo weight is not different in the Dlk1-null embryos, meaning that the Dlk1-null embryos and placentas are both reduced in weight to the same extent. Previous studies that induced IUGR by diet restriction in pregnant rats showed 20% reductions in both placental and embryo weights, which persists into later life 43. A recent study showed no IUGR in mice with a conditional deletion of Dlk1 in placental endothelial cells 44, suggesting that the reduced placental size in Dlk1-null animals is most likely a result of loss of Dlk1 in other embryonic cell types. It is also possible that the reduced placental size is a consequence of other defects in DLK1-dependent embryonic development, which also leads to IUGR.

An additional possibility to reconcile a smaller body size with only a mild GH content reduction is a defect in the ability of GH cells to secrete GH in response to GH secretagogues. Ultrastructural localisation of Dlk1 has been shown in the paranuclear rough endoplasmic reticulum and secretory vesicles 23, suggesting a possible involvement in hormonal secretion. However, in 6-week-old male Dlk1-null mice, we found no defect in the immediate response to acute challenge by both GHRP-6 and GHRH. There is an approximately 50% reduction in plasma GH concentration 15 min after administration of both GHRP-6 and GHRH, although this only reached significance after GHRH challenge (P = 0.035). The reduction is again not as severe as that seen in GH-M2 mice with normal growth 40. The organisation of the GH cell network has previously been found to be important for sustained calcium activity in response to GHRH stimulation 33. The reduction in plasma GH concentration at the 15-min time point would be consistent with the alteration in the organisation of GH cells reported by Puertas-Avendano *et al*. 23 affecting the coordinated secretion of GH. In addition, the larger reduction of GH release in response to GHRH stimulation may suggest differential effects of loss of Dlk1 on signalling pathways utilised by the GHRH-receptor (adenylate cyclase activation) 7 and the GHRP-6 stimulated ghrelin receptor (mitogen-activated protein kinase pathways) 45.

Dlk1-null mice were found to have mild reductions in GH contents during all stages in both sexes throughout life, from late embryonic development to adulthood. All other pituitary hormones were found to display mild reductions in an age- and sex-specific manner. It is possible that there is an interaction between the pituitary hormone deficiencies leading to reduced growth, especially from reduced TSH content. However, in mice with a similarly low TSH content 40, plasma thyroxine concentrations were not reduced, suggesting that the Dlk1 mutant may similarly not have reduced plasma thyroid hormone. The relative proportion of each hormone cell type was unchanged at 6 weeks and, because the total pituitary protein suggests no change in the total number of pituitary cells in Dlk1-null mutants, it is likely that the total number of each of the hormone cell types is unchanged. This suggests that it is the hormone content per cell that is reduced in Dlk1-null mutants. Since the Notch signalling pathway has a role in regulating hormone cell differentiation 11,12,15,46, a difference in the proportion of endocrine cells might have been expected; however, it is possible that other Notch ligands compensate for the loss of Dlk1 to allow correct specification of pituitary endocrine cells. It is unclear whether the reductions in pituitary hormone contents reflects a common role for Notch signalling in the regulation of gene expression in the different cell types of the pituitary, or whether this is a secondary effect of loss in nonpituatary tissues. However, a recent study with somatotroph specific deletion of Dlk1 with no effect on GH gene expression suggests that secondary effects from nonpituitary tissue is at least part of the cause of reduced hormone content 44.

Reduced pituitary FSH contents at all ages concurs with previous reports of reduced FSH immunoreactivity and mRNA content in the Dlk1-null mutant pituitary 23. Although we observe reductions in pituitary gonadotrophins, there is no noticeable defect in fertility or litter size, and the onset of puberty appears to be normal with mutant mice breeding normally at 6 weeks of age. Pituitary PRL content was unaffected in male Dlk1-null mice, which is consistent with the normal gene expression reported by Puertas-Avendano *et al*. 23, although that study reported a reduced PRL immunoreactivity. Our finding that pituitary GH content in adult animals is reduced with normal cell number is also different to the findings of Puertas-Avendano *et al*. 23 who found normal mRNA expression but a reduction in somatotroph cell number. The reason for these discrepancies between our measurements of hormone content and the GH expression and PRL immunoreactivity of Dlk1-null pituitaries found by Puertas-Avendano *et al*. 23 is unclear but may be a result of differences in the background strain used or age of mice examined.

Dlk1 has been previously shown to colocalise in mostly GH cells and only a small proportion of other hormone-producing cell types 22,23, which we have confirmed by immunohistochemistry. This would suggest that the effects of loss of Dlk1 on non-GH pituitary cells is indirect and may result from paracrine effects, either through Dlk1 itself or a secondary factor secreted by GH cells, on the other cell types. Dlk1 expression has also been described in the putative pituitary progenitor population expressing Sox2 and Sox9 24,25, although, in our studies, the majority of Sox2-positive cells are not Dlk1-positive, suggesting that only a subset of Sox2-positive cells express Dlk1. Because Notch signalling has been implicated in the regulation of endocrine differentiation during development 11,12,15,46, an alteration in the proliferation and differentiation of these progenitor cells could also lead to the pituitary phenotype of Dlk1-null mice. Although there is a complete loss of Dlk1 expression beginning in the embryo, we see no effect on the specification of cells into hormone-producing cell types in the developed pituitary gland, possibly suggesting a functionally redundant role of Dlk1 during the development of the developing Rathke's pouch with other Notch ligands. Because the model used in the present study leads to a global loss of Dlk1, however, it is also possible that the changes in all pituitary hormones (including GH) may be secondary to the loss of Dlk1 in other tissues. For example, the altered serum leptin found by Puertas-Avendano *et al*. 23 may have an impact on all hormones in the pituitary that could vary with age and sex. It is also possible that the alterations in embryonic development, demonstrated by altered embryo size and pituitary GH content, could result in altered programming of the pituitary hormone axes.

The data reported in the present study have shown an effect of loss of Dlk1 on all pituitary hormone axes, although without overt changes to normal physiological function. These minor pituitary phenotypes are likely to be secondary to loss of Dlk1 elsewhere in the body. These Dlk1-null mice may reflect human pathologies lacking expression of Dlk1 and suggest a minor contribution of the pituitary to pathological conditions, having implications in clinical considerations and treatments of human Dlk1-null conditions. This is supported by the description of a model with a conditional loss of Dlk1 in somatotrophs, which had no growth phenotype or change in GH gene expression 44. A conditional loss of Dlk1 in placental endothelial 44 also does not recapitulate the growth defects observed in the present study. Further work using this conditional allele of Dlk1 could prove crucial for unravelling the mechanisms that lead to the primary causes of the pituitary deficiencies.

## References

[1] Brinkmeier ML, Davis SW, Carninci P, MacDonald JW, Kawai J, Ghosh D, Hayashizaki Y, Lyons RH, Camper SA (2009). Discovery of transcriptional regulators and signaling pathways in the developing pituitary gland by bioinformatic and genomic approaches. Genomics.

[2] Carbajo-Perez E, Watanabe YG (1990). Cellular proliferation in the anterior pituitary of the rat during the postnatal period. Cell Tissue Res.

[3] Levy A (2002). Physiological implications of pituitary trophic activity. J Endocrinol.

[4] Lewinski A, Konopacki J, Pawlikowski M, Lewinska MK, Smith NK, Reiter RJ (1984). Effects of intraventricular injections of 6-hydroxydopamine on anterior pituitary cell proliferation. Anat Rec.

[5] Denef C (2008). Paracrinicity: the story of 30 years of cellular pituitary crosstalk. J Neuroendocrinol.

[6] Rizzoti K (2010). Adult pituitary progenitors/stem cells: from in vitro characterization to in vivo function. Eur J Neurosci.

[7] Mayo KE, Miller T, De Almeida V, Godfrey P, Zheng J, Cunha SR (2000). Regulation of the pituitary somatotroph cell by GHRH and its receptor. Recent Prog Horm Res.

[8] Wharton KA, Johansen KM, Xu T, Artavanis-Tsakonas S (1985). Nucleotide sequence from the neurogenic locus notch implies a gene product that shares homology with proteins containing EGF-like repeats. Cell.

[9] Kopan R, Ilagan MX (2009). The canonical Notch signaling pathway: unfolding the activation mechanism. Cell.

[10] Raetzman LT, Ross SA, Cook S, Dunwoodie SL, Camper SA, Thomas PQ (2004). Developmental regulation of Notch signaling genes in the embryonic pituitary: Prop1 deficiency affects Notch2 expression. Dev Biol.

[11] Zhu X, Zhang J, Tollkuhn J, Ohsawa R, Bresnick EH, Guillemot F, Kageyama R, Rosenfeld MG (2006). Sustained Notch signaling in progenitors is required for sequential emergence of distinct cell lineages during organogenesis. Genes Dev.

[12] Raetzman LT, Cai JX, Camper SA (2007). Hes1 is required for pituitary growth and melanotrope specification. Dev Biol.

[13] Monahan P, Rybak S, Raetzman LT (2009). The notch target gene HES1 regulates cell cycle inhibitor expression in the developing pituitary. Endocrinology.

[14] Himes AD, Raetzman LT (2009). Premature differentiation and aberrant movement of pituitary cells lacking both Hes1 and Prop1. Dev Biol.

[15] Raetzman LT, Wheeler BS, Ross SA, Thomas PQ, Camper SA (2006). Persistent expression of Notch2 delays gonadotrope differentiation. Mol Endocrinol.

[16] Takada S, Tevendale M, Baker J, Georgiades P, Campbell E, Freeman T, Johnson MH, Paulsen M, Ferguson-Smith AC (2000). Delta-like and gtl2 are reciprocally expressed, differentially methylated linked imprinted genes on mouse chromosome 12. Curr Biol.

[17] Schmidt JV, Matteson PG, Jones BK, Guan XJ, Tilghman SM (2000). The Dlk1 and Gtl2 genes are linked and reciprocally imprinted. Genes Dev.

[18] Abdallah BM, Ding M, Jensen CH, Ditzel N, Flyvbjerg A, Jensen TG, Dagnaes-Hansen F, Gasser JA, Kassem M (2007). Dlk1/FA1 is a novel endocrine regulator of bone and fat mass and its serum level is modulated by growth hormone. Endocrinology.

[19] Larsen JB, Jensen CH, Schroder HD, Teisner B, Bjerre P, Hagen C (1996). Fetal antigen 1 and growth hormone in pituitary somatotroph cells. Lancet.

[20] Tornehave D, Jensen CH, Teisner B, Larsson LI (1996). FA1 immunoreactivity in endocrine tumours and during development of the human fetal pancreas; negative correlation with glucagon expression. Histochem Cell Biol.

[21] Yevtodiyenko A, Schmidt JV (2006). Dlk1 expression marks developing endothelium and sites of branching morphogenesis in the mouse embryo and placenta. Dev Dyn.

[22] Nakakura T, Sato M, Suzuki M, Hatano O, Takemori H, Taniguchi Y, Minoshima Y, Tanaka S (2009). The spatial and temporal expression of delta-like protein 1 in the rat pituitary gland during development. Histochem Cell Biol.

[23] Puertas-Avendano RA, Gonzalez-Gomez MJ, Ruvira MD, Ruiz-Hidalgo MJ, Morales-Delgado N, Laborda J, Diaz C, Bello AR (2011). Role of the non-canonical Notch ligand DLK1 in hormone-producing cells of the adult male mouse pituitary. J Neuroendocrinol.

[24] Chen J, Gremeaux L, Fu Q, Liekens D, Van Laere S, Van Kelecom H (2009). Pituitary progenitor cells tracked down by side population dissection. Stem Cells.

[25] Fauquier T, Rizzoti K, Dattani M, Lovell-Badge R, Robinson IC (2008). SOX2-expressing progenitor cells generate all of the major cell types in the adult mouse pituitary gland. Proc Natl Acad Sci USA.

[26] Ansell PJ, Zhou Y, Schjeide BM, Kerner A, Zhao J, Zhang X, Klibanski A (2007). Regulation of growth hormone expression by Delta-like protein 1 (Dlk1). Mol Cell Endocrinol.

[27] Altenberger T, Bilban M, Auer M, Knosp E, Wolfsberger S, Gartner W, Mineva I, Zielinski C, Wagner L, Luger A (2006). Identification of DLK1 variants in pituitary- and neuroendocrine tumors. Biochem Biophys Res Commun.

[28] Gejman R, Batista DL, Zhong Y, Zhou Y, Zhang X, Swearingen B, Stratakis CA, Hedley-Whyte ET, Klibanski A (2008). Selective loss of MEG3 expression and intergenic differentially methylated region hypermethylation in the MEG3/DLK1 locus in human clinically nonfunctioning pituitary adenomas. J Clin Endocrinol Metab.

[29] Cheunsuchon P, Zhou Y, Zhang X, Lee H, Chen W, Nakayama Y, Rice KA, Tessa Hedley-Whyte E, Swearingen B, Klibanski A (2011). Silencing of the imprinted DLK1-MEG3 Locus in human clinically nonfunctioning pituitary adenomas. Am J Pathol.

[30] Moon YS, Smas CM, Lee K, Villena JA, Kim KH, Yun EJ, Sul HS (2002). Mice lacking paternally expressed Pref-1/Dlk1 display growth retardation and accelerated adiposity. Mol Cell Biol.

[31] Raghunandan R, Ruiz-Hidalgo M, Jia Y, Ettinger R, Rudikoff E, Riggins P, Farnsworth R, Tesfaye A, Laborda J, Bauer SR (2008). Dlk1 influences differentiation and function of B lymphocytes. Stem Cells Dev.

[32] Charalambous M, Ferron SR, da Rocha ST, Murray AJ, Rowland T, Ito M, Schuster-Gossler K, Hernandez A, Ferguson-Smith AC (2012). Imprinted gene dosage is critical for the transition to independent life. Cell Metab.

[33] Sanchez-Cardenas C, Fontanaud P, He Z, Lafont C, Meunier AC, Schaeffer M, Carmignac D, Molino F, Coutry N, Bonnefont X, Gouty-Colomer LA, Gavois E, Hodson DJ, Le Tissier P, Robinson IC, Mollard P (2010). Pituitary growth hormone network responses are sexually dimorphic and regulated by gonadal steroids in adulthood. Proc Natl Acad Sci USA.

[34] Kobayashi S, Wagatsuma H, Ono R, Ichikawa H, Yamazaki M, Tashiro H, Aisaka K, Miyoshi N, Kohda T, Ogura A, Ohki M, Kaneko-Ishino T, Ishino F (2000). Mouse Peg9/Dlk1 and human PEG9/DLK1 are paternally expressed imprinted genes closely located to the maternally expressed imprinted genes: mouse Meg3/Gtl2 and human MEG3. Genes Cells.

[35] Le Tissier PR, Carmignac DF, Lilley S, Sesay AK, Phelps CJ, Houston P, Mathers K, Magoulas C, Ogden D, Robinson IC (2005). Hypothalamic growth hormone-releasing hormone (GHRH) deficiency: targeted ablation of GHRH neurons in mice using a viral ion channel transgene. Mol Endocrinol.

[36] McGuinness L, Magoulas C, Sesay AK, Mathers K, Carmignac D, Manneville JB, Christian H, Phillips JA, Robinson IC (2003). Autosomal dominant growth hormone deficiency disrupts secretory vesicles in vitro and in vivo in transgenic mice. Endocrinology.

[37] Castrique E, Fernandez-Fuente M, Le Tissier P, Herman A, Levy A (2010). Use of a prolactin-Cre/ROSA-YFP transgenic mouse provides no evidence for lactotroph transdifferentiation after weaning, or increase in lactotroph/somatotroph proportion in lactation. J Endocrinol.

[38] Croteau S, Roquis D, Charron MC, Frappier D, Yavin D, Loredo-Osti JC, Hudson TJ, Naumova AK (2005). Increased plasticity of genomic imprinting of Dlk1 in brain is due to genetic and epigenetic factors. Mamm Genome.

[39] Ferron SR, Charalambous M, Radford E, McEwen K, Wildner H, Hind E, Morante-Redolat JM, Laborda J, Guillemot F, Bauer SR, Farinas I, Ferguson-Smith AC (2011). Postnatal loss of Dlk1 imprinting in stem cells and niche astrocytes regulates neurogenesis. Nature.

[40] Waite E, Lafont C, Carmignac D, Chauvet N, Coutry N, Christian H, Robinson I, Mollard P, Le Tissier P (2010). Different degrees of somatotroph ablation compromise pituitary growth hormone cell network structure and other pituitary endocrine cell types. Endocrinology.

[41] Ward RD, Stone BM, Raetzman LT, Camper SA (2006). Cell proliferation and vascularization in mouse models of pituitary hormone deficiency. Mol Endocrinol.

[42] Rossant J, Cross JC (2001). Placental development: lessons from mouse mutants. Nat Rev Genet.

[43] Woodall SM, Breier BH, Johnston BM, Bassett NS, Barnard R, Gluckman PD (1999). Administration of growth hormone or IGF-I to pregnant rats on a reduced diet throughout pregnancy does not prevent fetal intrauterine growth retardation and elevated blood pressure in adult offspring. J Endocrinol.

[44] Appelbe OK, Yevtodiyenko A, Muniz-Talavera H, Schmidt JV (2012). Conditional deletions refine the embryonic requirement for Dlk1. Mech Dev.

[45] Garcia A, Alvarez CV, Smith RG, Dieguez C (2001). Regulation of Pit-1 expression by ghrelin and GHRP-6 through the GH secretagogue receptor. Mol Endocrinol.

[46] Goldberg LB, Aujla PK, Raetzman LT (2011). Persistent expression of activated notch inhibits corticotrope and melanotrope differentiation and results in dysfunction of the HPA axis. Dev Biol.

